# Socializing social sampling models: The limits of explaining norm perceptions and biases with sampling from social circles

**DOI:** 10.1371/journal.pone.0286304

**Published:** 2023-06-02

**Authors:** Helge Giese, Janina A. Hoffmann

**Affiliations:** 1 Department of Psychology, University of Konstanz, Konstanz, Germany; 2 Heisenberg Chair for Medical Risk Literacy and Evidence-Based Decisions, Center for Anesthesiology and Intensive Care, Charité – Universitätsmedizin Berlin, Berlin, Germany; 3 Department of Psychology, University of Bath, Bath, United Kingdom; University of Glasgow, UNITED KINGDOM

## Abstract

People often overestimate the prevalence of unfavorable behavior. To explain these misperceptions, social sampling models propose that individuals infer the social norm from the behavior of their own social circle. We investigated this idea by asking a friendship network of college freshmen to report their own behavior and norm perceptions across eight domains at two timepoints (N = 104). Assessing this complete social network allows to directly test if sampling from the social circle shapes norm perception. Replicating previous findings, freshmen systematically misperceived the average social norm within their cohort. Yet, these misperceptions persisted even when individuals judged their own social circle, indicating that sampling from social circles does not fully explain normative biases. Moreover, cognitive modelling of norm perceptions suggested that individuals unlikely limited their search to their own social circle.

## Introduction

While the behavior and standards we observe in others affect our own behavior (e.g., [1,2]) individuals do not accurately judge the behavior of others and systematically misperceive these descriptive norms. A wealth of research demonstrates that individuals consider their own attitudes and behavior as more prevalent in the larger population [3,4], overestimate the prevalence of undesirable behavior [5,6] or characteristics [7–9], and underestimate the prevalence of favorable characteristics [10]. Attempts to correct these norm perceptions show only limited success [9,11,12]. This article contributes to a better understanding of how individuals establish social descriptive norms by scrutinizing current models of social norm perception.

### Why may people fail to perceive social norms accurately?

Three explanations for the misjudgment of descriptive social norms dominate the literature: motivational biases, cognitive incompetence, and sampling errors. Motivational explanations, like self-enhancement, are based on the observation that individuals view themselves and their own behavior more favorably compared to others (e.g., [8]). While one cannot rule out strategic underreporting of undesirable behavior, motivational biases frequently only describe the bias without explaining how people generate the social norm. In addition, both motivational biases and cognitive incompetence—the failure to recognize the ability of others [13]—cannot explain contrary findings such as viewing oneself less favorably than others (self-deprecation; e.g., [14]). Finally, explanations focusing on sampling error propose that individuals underrepresent frequent behaviors and overrepresent rare behaviors, similar to a regression-to-the-mean [15–19]. These sampling errors simultaneously predict self-enhancement and self-deprecation independent of the desirability of the behavior, and can even explain observations of underestimated dispersion of behavior in groups.

More recently developed *social* sampling models [20–22] add to this sampling approach by elucidating how people generate the descriptive social norm in response to their social surrounding. These models assume that people mentally represent their social circles and use these representations as a proxy to estimate the prevalence of a behavior in the population. According to these models, the interplay between individual sampling processes, such as retrieval from memory, and a social network in which persons with similar characteristics as the self are overrepresented (*homophily*, [23]) successfully explains how the distribution of characteristics in the population distorts descriptive norm estimates [20], or how people infer frequencies [24]. In addition, social sampling models identified further mechanisms that sustain the misperception of descriptive social norms. For instance, Lerman et al. [22] demonstrated that even if only popular group members demonstrate a certain behavior individuals should overestimate the prevalence of this behavior in the population (c.f., [6]). Taken together, social sampling models present a parsimonious, psychologically motivated, testable explanation of why people often fail to accurately judge their social environment.

Thus far, psychological studies on social sampling models have mainly relied, however, on individuals’ perception of their circle, that is self-reports, and have neglected whether this perception of the social circles actually matches the self-perception of its members. This simplification was previously justified by the argument that individuals perceive and judge the behavior of close others more accurately than the behavior of more abstract populations (e.g., [5,16]). Yet, self-reports may better align with friends’ behavior because individuals extend their social identity to one’s inner social circles rather than the population (e.g., [25,26]). In addition, asking individuals to provide the same reports for their own social circle and for the general population likely confounds the similarity of the social circle and the general population with the similarity in their measurement. Furthermore, the same memory failures and adjustment processes that distort norm perception in the general population should underpin the perception of the smaller social networks, as these mechanisms should be independent of the size of the judged group [15]. Therefore, self-reports of the social circle cannot provide a critical test of the idea that individuals sample from their social circle when inferring descriptive norms in the population. Rather, a coherent test requires to track the behavior of each individual as well as the behavior of their acquaintances—their social circle.

### The current study

The current study aims to scrutinize social sampling in a social network in which each individuals’ behavior and their social relationships are known. To observe a complete social network, we recruited a cohort of psychology freshmen, asked the students about their relationships to one another, about their own behavior (e.g., drinking frequency), and their perception of the corresponding descriptive norm in this network. Knowing the self-reported behavior of each person within the network allows us to infer the behavior of each individual’s social circle and to predict how each individual should have perceived the behavioral norm in the population if they based these norm perceptions on the behavior reported by their social circle. Moreover, if individuals broaden their social circle as time passes by, for a restricted social network one should expect that individuals should more accurately infer social norms at a later timepoint [6,20]. As such, a second time-point can be used both to cross-validate findings and to test to which extent an improved knowledge about the population will reduce the misperception of social norms.

Specifically, the current study tests whether and to what extent people preferably sample from their social circles to estimate the descriptive norm within the population by comparing these perceptions to the self-reported behavior of the individuals’ social circle. The assumption of sampling by social closeness leads to following expectations:

1. We expect to replicate the finding that people systematically misperceive the central tendency in a distribution of a behavior. If people use their social circle to estimate the population norm, biases in norm perception should depend on the skew in the distributions: If the distribution of a behavior is positively skewed in the population, those behaviors should be overestimated. If the distribution of a behavior is negatively skewed in the population, those behaviors should be underestimated.
2. If sampling from the social circle explains potential biases, we expect that biases in descriptive norm perception should disappear when individuals judge the prevalence of behavior in their own social circles, in particular when accounting for sampling from this social circle.
3. The perceived behavior of acquaintances should correlate positively with the acquaintances’ self-reported behavior indicating that people can accurately infer descriptive norms based on social observations.
4. Cognitive process models that restrict sampling to the social circle should describe individual norm perceptions more successfully than population-based accounts, that is, accounts that sample from the full population. These estimation strategies should be stable over time.

## Methods

### Participants and procedure

All 112 first-year Psychology students of the University of Konstanz, who started their studies that month, were invited to take part in the study in an introductory session in the last week of October 2018. Questionnaires were sent out via personalized e-mails for the initial assessment in the first two weeks of November (t0) and for the final assessment in the first two weeks of May (t1). In total, 108 students (80 females; *M*_Age_ = 21.4, *SD* = 0.5) completed the questionnaire at t0 and received 5€ or course credit as compensation. 104 of the initial 108 participants completed the second questionnaire at t1 and received 15€ or course credit (see [6] for a similar procedure). While the sample size was constrained by the total group size of 112, the obtained sample size should be sufficient to find medium-sized effects (*r =* .34) with a power of.95 [27].

Participants gave written informed consent when registering for the study. At the beginning of the study, participants completed demographic and trait questionnaires at both time points. Afterwards, participants were asked to report their own behavior and to estimate how often their acquaintances and the entire cohort engaged in a particular behavior (norm estimations). The order of self-reports and norm estimations (separately for the population and their acquaintances) was randomized between participants. Finally, we asked each individual about their social circle (i.e., their relationships to the other group members).

The study reported in this article was not formally preregistered. The study was conducted in accordance with the declaration of Helsinki with written informed consent and approved by the University of Konstanz ethics board. Anonymized data to reproduce the results can be obtained at https://osf.io/x7sek with the doi 10.17605/OSF.IO/X7SEK.

### Measures

#### Self-reported behavior

At both time points, all participants indicated for the last month a) the total number of their friends, b) their net income (in €), c) the average number of alcoholic drinks per week, d) the average number of hours per week spent studying outside university, e) the experienced stress while studying, f) frequency of conflicts with others, and g) the frequency of meat and sausage consumption. Participants answered a) to d) in an open text field that allowed numbers only and rated e) to g) on 5-point rating scales (“*not at all*”—“*completely”* for experienced stress; “*never”—*“*(almost) daily*” for frequencies). The behaviors were selected to reflect different distributions based on similar samples [6] or directly to replicate social sampling studies [20] and their scaling is presented in Table 1.

**Table 1:** Descriptive statistics for all rated behaviors denoting over- and underestimation dependent on the skew of the population distribution.

	Alcohol (number standard drinks/week)		Friends (number)		Conflicts (days/month)		Income (€/month)		Study time (in h/week)		Study Stress (1 not at all 5 completely)		Meat Consumption (days/month)	
T0														
Skew in population	1.41		1.62		5.32		4.68		–0.6		0.38		1.42	
	M	SD	M	SD	M	SD	M	SD	M	SD	M	SD	M	SD
Acquaintances	5.56^a^	4.04^a^	7.41^a^	5.07^a^	1.47^a^^+^	3.00^a^^+^	630.2^a^^+^	303.7^ac^^+^	11.07^a^^+^	4.83^a^^+^	2.50^a^^+^	0.89^a^^+^	6.48^a^	7.33^a^
Friends	5.34^a^	2.44^b^^+^	7.68^a^	3.12^b^^+^	1.23^a^^+^	1.24^b^^+^	623.2^a^	192.4^b^^+^	10.85^a^	3.15^b^^+^	2.49^a^	0.51^b^^+^	6.45^a^	5.30^b^^+^
Perceived population	7.72^b^^+^	4.15^a^	7.11^a^	3.80^b^^+^	2.31^b^^+^	2.92^a^^+^	669.7^a^	315.4^c^^+^	7.93^b^^+^	3.97^c^^+^	2.47^a^	0.81^a^^+^	8.55^b^^+^	7.92^a^^+^
Perceived acquaintances	7.51^b^^+^	3.32^c^^+^	7.20^a^	3.55^b^^+^	1.58^a^	1.74^b^^+^	632.2^a^	262.1^a^^+^	7.04^c^^+^	3.38^b^^+^	2.41^a^	0.65^c^^+^	7.12^a^	5.82^b^^+^
F-value	34.9***	34.8***	0.9	29.5***	6.8**	19.3***	1.6	24.1***	69.4***	27.1***	0.6	41.7***	8.1***	15.9***
Partial η^2^	.275	.279	.010	.247	.069	.177	.017	.211	.430	.231	.007	.317	.081	.150
Corrected population	5.32	4.04	7.13	5.08	1.54	3.61	648.2	358.5	10.74	5.06	2.56	0.94	6.54	7.29
Skew in population	2.59		2.55		5.31		1.68		1.69		0.27		1.64	
	M	SD	M	SD	M	SD	M	SD	M	SD	M	SD	M	SD
Acquaintances	4.38^a^^+^	3.86^a^	8.33^ab^^+^	4.14^a^	1.58^a^	3.14^a^^+^	737.0^a^^+^	376.1^a^	5.93^a^^+^	4.89^a^^+^	2.40^a^^+^	0.92^a^^+^	5.18^a^	6.29^a^
Friends	4.31^a^	2.50^b^^+^	8.67^b^^+^	3.19^b^^+^	1.22^a^^+^	1.64^b^^+^	731.1^ab^	255.4^b^^+^	5.83^a^	3.76^bc^^+^	2.48^a^	0.70^b^^+^	5.33^a^	4.52^b^^+^
Perceived population	7.28^b^^+^	4.09^a^^+^	7.49^a^^+^	3.87^ac^^+^	2.22^b^^+^	3.28^a^	684.5^b^	324.6^c^^+^	7.94^b^^+^	4.04^c^^+^	2.47^a^	0.85^a^^+^	8.87^b^^+^	7.19^c^
Perceived acquaintances	6.25^c^^+^	3.26^c^^+^	7.47^a^^+^	3.62^bc^^+^	1.72^ab^	2.08^c^^+^	683.3^b^	273.0^b^^+^	7.16^c^^+^	3.55^b^^+^	2.37^a^	0.75^b^^+^	5.67^a^	5.27^b^^+^
F-value	64.9***	24.0***	6.8**	9.6***	4.8**	23.8***	6.2**	19.8***	20.1***	15.2***	1.2	14.6***	27.0***	19.9***
Partial η^2^	.394	.193	.064	.088	.046	.192	.058	.165	.168	.132	.012	.127	.212	.166
Corrected population	4.14	3.70	8.08	4.24	1.63	3.65	701.9	368.9	6.20	5.24	2.45	1.01	5.38	6.52

Different superscripts per column indicate (Sidak-corrected) significant differences in the post-hoc tests of the ANOVAs testing for differences between all four groups: Acquaintances, friends, perceived population, and perceived acquaintances for each behavior at each time point. The degrees of freedom for the F-tests of these ANOVAs are at t0 M: *F*(3,276), *SD*: *F*(3,270) and at t1 *M*: *F*(3,300), *SD*: *F*(3,300).

**p_Greenhouse- Geisser_ < 0.01

***p_Greenhouse-Geisser_ < 0.001.

^+^Significantly different from the binned population estimate (corrected population) in one-sample t-tests at each time point for each behavior.

#### Norm estimations

**Fig 1:**
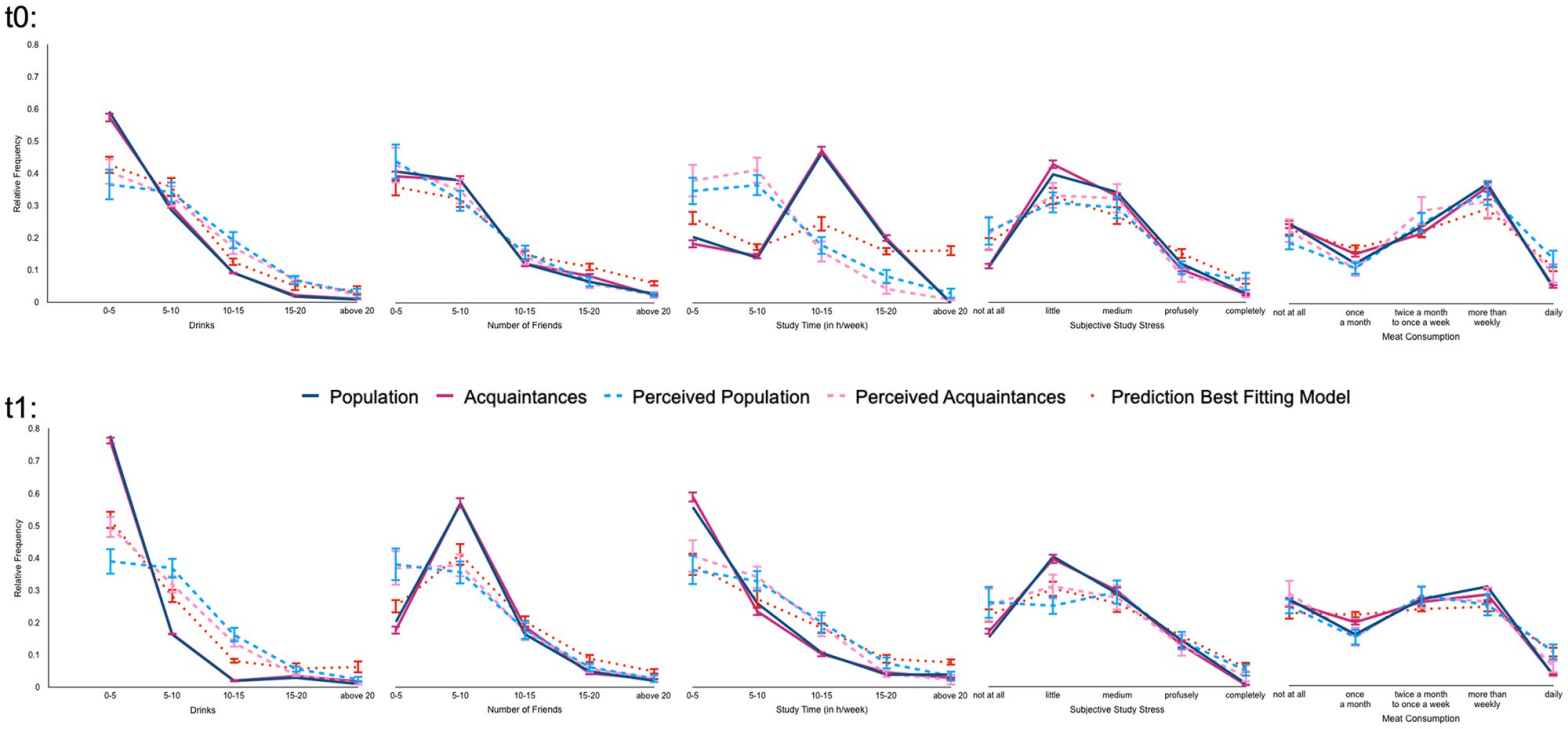
Relative frequency distributions of selected behaviors for t0 (upper panel) and t1 (lower panel). Bars represent 95%CI. The self-reports of the population (solid blue) and acquaintances (solid purple) systematically differ from their perceptions (dashed). The No Social Circle model is the best-fitting model of our cognitive modelling approaches fitting population perceptions (dotted red).

At both time points, participants estimated the distribution of the same seven behaviors in the population (i.e., their entire cohort) and among their acquaintances. For each behavior and social group (population or acquaintances), they estimated separately what proportion of individuals (in percentage points) engaged in these behaviors. For the acquaintance distribution estimation, participants were asked to consider students in their cohort as acquaintances, if they had at least one long conversation within the past half-year period and thus knew them better than sight [16]. To simplify estimations, participants drew both distributions using an online tool which forced percentage point estimates to add up to 100 across five response categories (all response categories are reported in Fig 1). We determined these five response categories from previous data on self-reported behavior with each response category either reflecting the scale points of the rating scales or bins of 5 units (starting with “*up to 5*” units and ending with “*more than 20*” units in increments of 5 units; e.g., for income, a unit consisted of 100€). For instance, participants estimated what percentage of individuals in their cohort drank up to 5, 5 to 10, 10 to 15, 15 to 20, and 20 or more standard alcoholic drinks. Participants were reminded before the task that their cohort consisted of 112 people.

#### Social circle

To assess the social circle, each individual rated their relationship with all other participating students by indicating in a randomized name list which person they “*knew better than sight*”, “*befriended*”, “*liked*”, and “*disliked*”. Friendship was defined as “*a person you feel emotionally close to*, *with whom you discuss personal matters*, *and whom you can ask for help*”, acquaintanceship (knowing better than sight) was defined as “*talked to this person within the past half-year period at least once for a longer time*”. Each name in the name list was presented together with a picture of that person to facilitate identification. At each time point, inconsistent ratings of one individual were eliminated (e.g., when a person was selected as “liked and “disliked at the same time in one assessment; t0/t1: 4 instances, see also [6]).

As expected, we find that students on average have a bigger social network at t1 than at t0 (friends: t0: *M =* 4.73, *SD =* 4.27; *t1*: *M =* 7.52, *SD =* 5.14; difference: *t*(103) = 7.58, *p* < 0.001, *d* = 0.74; acquaintances: t0: *M =* 34.96, *SD =* 15.67; *t1*: *M =* 38.43, *SD =* 15.77; difference: *t*(103) = 2.30, *p* = 0.024, *d* = 0.23). In sum, this nomination procedure yielded a) a measure of acquaintances as descriptive comparison standard which included all individuals known better than sight, and b) a measure of closeness, that is, friendships and acquaintance status as indicated by participants for modelling purposes.

## Results

### Group over- and underestimation in the first year of university

Replicating previous findings (e.g., [5,8,16]), we find that how first-year psychology students perceive the behavior of their peers systematically differs from the self-reported behavior of these peers. To compare self-reported behavior to norm perceptions, the continuous self-reports were discretized into the same categories as perceptions. Category means (e.g., 2.5, 7.5, 12.5, 17.5, 22.5 for alcoholic beverages) were used to compute both standard deviations and averages for self-reported and perceived behavior. We then compared average self-reported behavior to the average of perceived norm estimations in one-sample t-tests to test for biases in the perception of the population.

As Table 1 suggests, participants overestimate the mean perceived descriptive social norms for most behaviors compared to the self-reports of the population. For instance, they overestimate how many standard units of alcohol other people drink compared to the self-reported population mean (t0: *t*(107) = 8.49, *p* < 0.001, *d* = 0.82; t1: *t*(103) = 13.08, *p* < 0.001, *d* = 1.28).

Yet, the same individuals underestimate how many hours other students reported studying in the beginning of the academic year (t0: *t*(107) = –9.19, *p* < 0.001, *d* = –0.88; see also Fig 1), but overestimate the same behavior at t1 (*t*(103) = 5.34, *p* < 0.001, *d* = 0.52), reversing the direction of the effect. This pattern cannot be well accounted for by social desirability in self-reports or motivational biasing of group perceptions: If participants on average perceive the ideal student as hard-working and consider themselves “better than average”, they should not overestimate others’ study time at t1. In addition, one would assume that the individual valuation or desirability of the behavior should be rather stable, so that that a change from under- to overestimation is hard to explain by these approaches.

Instead, the degree of over- and underestimation varies systematically with the distribution of the behavior in the population, as expected by sampling accounts of estimation errors ([15]; Table 1). Across both time points, participants systematically overestimated the perceived norm for the 8 behaviors with a positive skew above 0.5 (e.g., drinking behavior), whilst they underestimated the perceived norm for the one behavior with a negative skew below -0.5. Norm perception was unbiased for the 2 behaviors without a large skew. In addition, participants systematically underestimate how much the behavior varies among their acquaintances, as indicated by smaller standard deviations for acquaintances’ perceptions than for acquaintances self-reports for most behaviors (Table 1). Taken together, this pattern better aligns with sampling accounts of biased norm perceptions, that are concerned with the distribution of the behavior, rather than motivational, social desirability, or cognitive incompetence accounts.

### Does the behavior of one’s social circle serve as a proxy for perceived social norms in the population?

#### The role of the social circle for over- and underestimation

If individuals can more accurately perceive and report the behavior of their social circle than the behavior of the population as a whole, then we expected biases in norm perception to disappear when comparing self-reports of acquaintances to the perceived norm in acquaintances. Yet, the same pattern of over- and underestimated perceived norms prevails: Across all behaviors and at both timepoints, the average of the perceived distributions deviated from the averages of acquaintances’ and friends’ self-reports (repeated-measures MANOVA t0: Pillai’s trace *V* = 0.620, *F*(21, 816) = 10.127, *p* ≤.001, η^2^_p_ = .207; t1: Pillai’s trace *V* = 0.638, *F*(21, 888) = 11.424, *p* ≤.001, η^2^_p_ = .213): In most cases of bias, the means of the binned reports by friends and acquaintances were also different from the perceived means for the population and—to lesser extent—also to the perceptions of acquaintances. This illustrates biases prevail even when comparing perceptions directly to individual social samples and thereby accounting for oversampling of popular people’s behavior (Table 1). This suggests that sampling from social circles in the environment does not fully explain normative biases.

#### Overall accuracy of the perception of acquaintances

Our study aimed to shed light on the question of whether people draw upon their social circle to predict the norm in the general population. At first sight, this social circle assumption seems to hold: Fig 1 displays that—on average—students perceive the behavior of their acquaintances as highly similar to how they perceive the population. In addition, mean norm estimates for acquaintances correlate strongly with average norm estimates for the population across all behaviors (S1 Table in S1 File), suggesting a relation between one’s social circle and social norm perception.

Yet, how individuals perceived the behavior of their acquaintances did not reflect their self-reported behavior in correlative measures. The average behavior reported by ones’ acquaintances only slightly correlated with how the individual perceived the mean behavior of their acquaintances for most behaviors (*MD*_*r*_ = 0.13; S1 Table in S1 File). Generally, the perception of acquaintances deviates on average per category more than 10% from the acquaintances’ self-reports (average mean absolute error *MAE*_*t0*_ = 10.33% and *MAE*_*t1*_ = 11.33%). These correlations (S1 Table in S1 File) are too low and the errors are too high to presume “that people have good knowledge of their immediate social circles” [20]—even granting some noise. In fact, the perceptions of others correlate mainly with an individual’s self-reports (*MD*_*r*_ = 0.33; 0.32, when accounting for homophily; S1 Table in S1 File) and not with external behavioral cues indicated by their network (*MD*_*r*_ = 0.13; S1 Table in S1 File). This result suggests that people rather project their own behavior on their social circle and by extension on the population than infer the population norm from socially close individuals (see also S1 Fig in S1 File).

#### Modelling a potential sampling process based on social distance

Thus far, we tested the social sampling hypothesis by evaluating how well norm perceptions match actual, self-reported behavior on an aggregated level. Yet, social sampling models aim to describe how single individuals infer the social norm from observing the behavior of others. Previous social sampling approaches have explicitly formulated how individuals form social norms by retrieving their social circle’s behavior from memory [20]. Indeed, what individuals know about their social circle predicts their social norm estimates quite well in our study and fares better than predicting social norm estimates from the self-reported behavior of the whole student population, albeit without necessarily needing to adjust for homophily or memory errors (see S1 File for details on the comparison). Assessing acquaintances’ real behavior allowed us to further investigate sampling from the real social environment: How far does sampling from the actual social circle in the environment explain the discrepancy between acquaintances’ reported behavior and perceived norms?

To test this question, we introduce a *sampling by distance assumption*. When individuals think of their social circle, we assume that individuals sample more likely those persons from the general population who are in a closer social relationship with them. Friends are thus more likely to be considered in the social circle than acquaintances. We introduce this sampling by distance assumption by extending the Social Sampling Model [20] formally with the social circle parameter γ. This parameter measures the size of the social circle, that is, what percentage of the population the person still considers from the general population as belonging to their social circle, when inferring the norm. Introducing this parameter is advantageous because some individuals may only think of their friends when inferring social norms, whereas others may explicitly consider the behavior of the “unknown” other. Previous approaches were unable to estimate this social circle parameter because the actual self-reported behavior of the social circle and their relationship to other persons was unknown. Knowing both reported behaviors and relationships thus allows us to directly test the *sampling by distance* assumption.

Formally, in the extended Social Sampling Model we predict how individuals perceive the social norm in the population—their population norm estimates—from the self-reported behavior of all other persons—the population distribution. The social circle parameter γ determines which other individuals from the population the person accesses depending on their social distance: only the γ socially closest individuals (sorted by category in the order: individual, friends, acquaintances, rest; randomized within category) remain in the sample. The social circle parameter γ thus provides the *i*^th^ percentile *pct*_*i*_ of the least socially close individual in the population that is still included in the social circle.

$$A_{SC}=1,\;\text{if}\;{\text{pct}}_{\text{i}}\leq \gamma ,\;\text{else}\;A_{SC}=0$$

where the social circle activation *A*_*SC*_ reflects if the person is included in the social circle. If individuals only sample all their friends, this parameter should take the value of γ = .09 because on average students consider 8.2% of the other students as their friends at T1 (SD = 5.0%). If individuals also sample their acquaintances, it should take value of.38 because on average students consider 37.5% of the other students as their acquaintances (SD = 15.1%).

In this social circle, those similar to the judging individual should be overrepresented under conditions of homophily. According to Galesic et al. [20], individuals adjust for this assumed overrepresentation in their evaluation and restrict the social circle further to individuals least similar to themselves, with behavioral similarity determined as the absolute distance to their own behavior within a category. Accordingly, the behavioral adjustment *ρ* determines the i^th^ percentile (*pct*_*i*_) of least behaviorally similar individuals that remain in the evaluation sample:

$$A_{RI}=1,\;\text{if}\;{\text{pct}}_{\text{i}}\leq \rho ,\;\text{else}\;A_{RI}=0$$

where the evaluation activation *A*_*RI*_ reflects if the person belongs to the evaluation sample.

In a final step, people attempt to retrieve individuals from this evaluation sample who belong to the target category *C*, but random memory errors may prevent retrieval with the probability α. Accordingly, the estimated proportion of the target category *C* within the reference class *R*, *p*(*C*|*R*), depends on the memory error (α), the activation of a specific individual of category *C* (*A*_*CI*_), the social circle activation of individual *i* (*A*_*SC*_), and the evaluation activation *A*_*RI*_:

$$p(C|R)=\frac{\sum_{i=1}^{n}\alpha \times A_{CI}\times A_{RI}\times A_{SC}}{\sum_{i=1}^{n}A_{RI}\times A_{SC}}$$

When predicting the probability of several response categories, the model assumes that individuals predict first the probability of the highest-frequency category and end their prediction with the lowest-frequency category to which missing instances are surcharged.

We implemented this extended Social Sampling Model with free parameters for all three processes (social sampling parameter γ, behavioral adjustment ρ, and memory error α, S1 File for details). We estimated all models using maximum likelihood estimation assuming a normally distributed error and therefore estimated an additional parameter for the standard deviation σ around the models’ predictions. We compared the full model to nested versions that restricted either one, two, or all three parameters (models are named by the parameter they include or omit) using two measures based on the Bayesian Information Criterion (BIC, [28]): BIC weights, BIC_w_, that provide the posterior probability of each model given the data [29] and the Bayes Factor, *BF*, the likelihood of one model M0 compared to an alternative model M1 ([30,31]; see S1 File). We also calculated how many individuals can be classified to one of the models given that the BIC_w_ is above.5, that is having a posterior probability of the model of more than.50.

At both timepoints, the extended Social Sampling Model with three parameters fares quite well and explains a higher amount of variance than the base rate population sampling model (see Table 2 for model fits and parameter estimates). This full model proposes that individuals think about γ = 40% of their socially closest peers (*SD* = 38%) when judging the social norm. Thus, the estimated size of their retrieved social circle matches—on average—the percentage of peers they perceive as acquaintances at t0 (33.3%, *SD* = 14.5%). Within this retrieved social circle, students base their norm perceptions only on those ρ = 56% of their peers who show dissimilar behavior to themselves. Finally, they may correctly observe and recall the behavior of others’ in α = 56% (*SD* = 32%) out of all estimates.

**Table 2:** Model fits and mean parameter estimates for each model for timepoint t0 and t1 predicting perceived population distributions based on the self-reported distributions of the population.

	T0								
Models	R^2^	RMSD	BIC	BIC_w_	CL	α	γ	ρ	σ
Base rate	0.66 (0.14)	19.3 (5.1)	253 (14)	.16 (.20)	11	—	—	—	21.6 (5.7)
Memory bias	0.67 (0.14)	18.6 (5.0)	254 (14)	.06 (.10)	2	.95 (.06)	—	—	20.8 (5.6)
Social circle	0.71 (0.12)	17.8 (4.5)	252 (13)	.13 (.18)	8	—	.57 (.42)	—	19.9 (5.0)
Behavior adjustment	0.68 (0.14)	18.7 (5.0)	255 (14)	.05 (.06)	0	—	—	.14 (.30)	21.0 (5.6)
No memory bias	0.72 (0.12)	17.6 (4.5)	255 (14)	.04 (.07)	0	—	.53 (.41)	.13 (.26)	19.6 (5.0)
**No social circle**	**0.76 (0.12)**	**16.0 (4.5)**	**249 (15)**	**.34 (.33)**	39	**.47 (.32)**	**—**	**.62 (.34)**	**17.9 (5.0)**
No behavior adjustment	0.72 (0.12)	17.2 (4.5)	253 (14)	.08 (.15)	5	.92 (.09)	.56 (.41)	—	19.2 (5.0)
Full model	0.77 (0.11)	15.4 (4.2)	250 (15)	.14 (.19)	6	.56 (.32)	.40 (.38)	.56 (.36)	17.2 (4.8)
	T1								
	R^2^	RMSD	BIC	BIC_w_	CL	α	γ	ρ	σ
Base rate	0.66 (0.15)	18.8 (5.1)	252 (14)	.11 (.18)	7	—	—	—	21.0 (5.7)
Memory bias	0.67 (0.15)	17.6 (4.9)	251 (15)	.04 (.07)	1	.93 (.07)	—	—	19.7 (5.5)
Social circle	0.72 (0.14)	17.0 (4.5)	249 (14)	.12 (.19)	7	—	.60 (.41)	—	18.8 (5.1)
Behavior adjustment	0.67 (0.15)	18.2 (5.0)	253 (14)	.04 (.10)	2	—	—	.16 (.32)	20.3 (5.5)
No memory bias	0.72 (0.14)	16.7 (4.6)	251 (15)	.04 (.07)	1	—	.57 (.40)	.15 (.28)	18.6 (5.2)
**No social circle**	**0.78 (0.13)**	**14.4 (4.7)**	**243 (17)**	**.35 (.35)**	38	**.40 (.33)**	**—**	**.68 (.34)**	**16.1 (5.2)**
No behavior adjustment	0.74 (0.13)	15.8 (4.5)	248 (15)	.09 (.17)	5	.90 (.11)	.66 (.38)	—	17.5 (5.2)
Full model	0.80 (0.13)	13.7 (4.5)	243 (16)	.21 (.27)	16	.46 (.34)	.39 (.34)	.64 (.34)	15.3 (5.0)

Standard deviations in parenthesis. α = memory bias (fixed: α = 1), γ = social circle parameter (fixed: γ = 0), ρ = behavioral adjustment (fixed: ρ = 0), σ = normally distributed error. The best fit according to BIC_w_, Bayesian Information Criterion weight, is bolded. CL denotes classifications to a 345 model, using a BIC_w_ >.5.

Yet, it does not clearly outperform the No Social Circle model omitting the assumption that individuals preferably sample peers from their own social circle (t0: *BF*_10_ = 0.58, $\bar{\Delta {\text{BIC}}_{\text{10}}}=1.1$, *SD* = 4.3; t1: *BF*_10_ = 0.8, $\bar{\Delta {\text{BIC}}_{\text{10}}}=0.4$, *SD* = 5.8; with No Social Circle model as the null hypothesis). This No Social Circle Model largely captured people’s average norm perceptions well (t0: *R*^2^ = 0.76, *SD* = .12; t1: *R*^2^ = 0.78, *SD* = .13) with *BF*s suggesting modest to strong evidence for the No Social Circle model compared to the population distribution and the base rate model (t0: *BF*_10_ = 7.4, $\bar{\Delta {\text{BIC}}_{\text{10}}}=-4.0$, *SD* = 9.1; t1: *BF*_10_ = 89.0, $\bar{\Delta {\text{BIC}}_{\text{10}}}=-9.0$, *SD* = 12.3; with No Social Circle model as the alternative hypothesis). It proposes that students base their norm perceptions—on average—only on those ρ = 62% of their peers (t1: ρ = 68%) who behave the least similar to themselves and recall the corresponding behavior correctly in α = 47% (t1: α = 40%) of the retrieval attempts.

Fig 1 illustrates how the No Social Circle Model (dotted red line) on average captures participants’ norm perceptions (dashed lines; perceived population in pink; perceived social circle in light blue) better than the population distribution (solid blue line) or the acquaintances’ distribution (solid red line). Similar to participants’ perceptions, the No Social Circle model, for instance, overestimates at t0 how many drinks other persons consume or how much study stress they experience. The No Social Circle model predicts participants’ norm perceptions for acquaintances quite well, too. Indeed, the parameter values of the No Social Circle models can be used to predict how participants perceive the distribution of their acquaintances’ behavior (*R*^2^_t0_ = .67, *SD*_t0_ = .14, *R*^2^_t1_ = .72, *SD*_t1_ = .15) highlighting that people may engage in similar strategies when judging norms within the population and their social circle. Taken together, similar cognitive processes may underpin misperceptions of social norms in social circles and the population, but social sampling of socially close others does not constitute a crucial component within sampling models, replicating descriptive results.

## Discussion

It is no secret that individuals often misperceive which behaviors other individuals typically engage in, or which traits characterize the majority (e.g., [5,8,16]). Studying an evolving friendship network allowed us to independently observe the behavior of socially close others, the individual perception of their behavior, and the perception of the whole peer group and therefore to directly test whether social closeness drives norm (mis-)perceptions.

First, we replicate that people are overly optimistic in positively skewed environments, but display self-deprecation in negatively skewed environments, a finding that can hardly be explained by motivational approaches. Interestingly, we also found a tendency to underestimate dispersion. These environmental dependencies are neglected by traditional motivational and cognitive accounts but can be parsimoniously explained by sampling accounts of norm perception [15–19]. In line with these findings, computational models that postulated cognitive explanations, such as adjusting for overrepresenting highly similar individuals and retrieval failures [20], described norm perceptions of the population relatively well.

Second, in line with social identity theory [25], students perceived the central tendency in the behavior of the population as more distant to themselves compared to the central tendency in the behavior of their acquaintances, that is their social circle (e.g., [5,16]). The classic social sampling account of Galesic et al. [20] also captured this generalization process from acquaintances to population by postulating both a similarity correction and a memory process that normalize the perception of acquaintances (see also S2 Table in S1 File).

Third, however, it seems that preferably sampling socially close individuals from the environment itself is insufficient to explain apparent misperceptions of group behaviors. Individuals perceive both social circle and population distributions as less skewed and smoother than the distributions of self-reports actually are, even when compared to a sampling-corrected distribution (see also [6]). Possibly, misperceptions partly originate from anchoring on one’s own behavior and adjusting the reported group norm accordingly. Furthermore, individuals do not even correctly perceive their own social circle, as indicated by small correlations between the self-report of the circle and its perception. In line with these descriptive observations, models of norm perception that predicted an individuals’ norm perception by sampling based upon social closeness from others’ self-reports were unable to conclusively predict these norm perceptions better.

All in all, a good understanding of how these misperceptions emerge beyond intangible motivational explanations is still missing. While projective processes seem to play a role in group perceptions, as also indicated by high self-norm perception correlations even after correcting for homophily (e.g., [3,6,32]), they cannot explain biases by the nature of their effects. One essential part of the bias may already be introduced by aggregating information about the individuals to group perceptions [7]—also pointed at by the role of non-normal distributions for biases.

### Limitations

One may argue that sampling from one’s social circle is more suitable for inferring social norms in large and diverse social groups in which the individual is unable to meet all group members, but is less applicable to our smaller and constrained study population. However, social circle models did not fare better at describing norm perceptions at baseline, although our participants only knew a few other group members at that timepoint. Still, investigations with larger, broader populations may be warranted.

Furthermore, as any objective standards are missing (generally, when applying Social Sampling Models, also in Galesic et al. [20]), systematic biases could also be introduced in self-reports, for instance due to social desirability. However, for example, the change in the direction of bias for studying time across measurement points is a pattern that would be implausible as a social desirability effect. Moreover, since social desirability pertains mainly to the estimates of central tendency, it does not account for the low associations between perceptions and self-reports.

As a further caveat, although the estimated individual parameters of the tested models generalized from group norm perceptions to acquaintances, they were not particularly stable across time points (e.g., BIC weights for the No Social Sampling model at time point 0 do not correlate with its BIC weights at timepoint t1 *r* = .04; S3 Table in S1 File). This result implies that sampling processes likely vary depending upon situational factors with the actual strategy applied, for instance, the neglect of similar others, being constrained by the task at hand. Another explanation for the instability of the sampling models may be that the perception of descriptive norms is less dependent on transient self-reported behaviors, but inferred from more stable cues [6].

Future research may seek to further understand which processes moderate the pathway from observing the behavior of (socially close) others to inferring the group norm. Cognitive sampling models provide a good starting point in this endeavor as they allow us to specify and test hypotheses about the underpinning processes and bridge the gap from behavior to perception.

## Supporting information

S1 File Online supplemental material. (PDF)
