# Supplementary material for: Socializing social sampling models: The limits of explaining norm perceptions and biases with sampling from social circles
Source: PLoS One. 2023 Jun 2;18(6):e0286304. doi: 10.1371/journal.pone.0286304 (PMC10237398; doi:10.1371/journal.pone.0286304)
Supplement: S1 File — (PDF) [file pone.0286304.s001.pdf]

# Online Supplemental Material

**S1 Table.** *Correlations of self-reports, self-reported norms, and perceived norms.*

| | **Alcohol (standard drinks)** | | **Friends (number)** | | **Conflicts (day/month)** | | **Income (€/month)** | | **Study time (in h)** | | **Study Stress (1-5)** | | **Meat Consumption (days/month)** | |
|---|---|---|---|---|---|---|---|---|---|---|---|---|---|---|
| | t0 | t1 | t0 | t1 | t0 | t1 | t0 | t1 | t0 | t1 | t0 | t1 | t0 | t1 |
| Correlations | | | | | | | | | | | | | | |
| Self-friend | 0.44*** | 0.32** | 0.10 | 0.04 | 0.00 | -0.04 | 0.00 | 0.28** | -0.04 | -0.09 | 0.31** | 0.17 | 0.13 | -0.01 |
| Self- acq. | 0.29** | 0.23* | 0.22* | 0.07 | -0.13 | -0.03 | -0.09 | 0.23* | -0.01 | 0.03 | 0.14 | 0.12 | 0.16 | 0.27** |
| Acq.-perc. acq. | 0.33*** | 0.16 | 0.27** | 0.10 | -0.08 | 0.04 | 0.05 | 0.20* | 0.01 | 0.06 | 0.07 | 0.15 | 0.32*** | 0.30** |
| Self-perc. acq. | 0.50*** | 0.59*** | 0.41*** | 0.53*** | 0.34*** | 0.00 | 0.19 | 0.29** | -0.12 | 0.31** | 0.49*** | 0.53*** | 0.22* | 0.18 |
| Self-perc. pop. | 0.48*** | 0.46*** | 0.44*** | 0.48*** | 0.25** | 0.10 | 0.34*** | 0.28** | -0.18* | 0.30** | 0.56*** | 0.51*** | 0.08 | 0.10 |
| Perc. acq.-perc. pop. | 0.73*** | 0.59*** | 0.67*** | 0.69*** | 0.81*** | 0.29** | 0.74*** | 0.88*** | 0.57*** | 0.76*** | 0.85*** | 0.76*** | 0.61* | 0.43*** |
| Partial Correlation (corrected for acquaintances' behavior) | | | | | | | | | | | | | | |
| Self- perc. acq. | 0.45*** | 0.57*** | 0.37*** | 0.53*** | 0.33*** | 0.00 | 0.19* | 0.26** | -0.12 | 0.31** | 0.48*** | 0.52*** | 0.19 | 0.11 |
| Self-perc. pop. | 0.43*** | 0.46*** | 0.42*** | 0.47*** | 0.25** | 0.10 | 0.35*** | 0.25* | -0.18 | 0.30** | 0.56*** | 0.50*** | 0.06 | 0.08 |
| Coleman Index | | | | | | | | | | | | | | |
| friends | 0.26 | 0.26 | -0.01 | -0.05 | 0.01 | 0.15 | 0.15 | -0.07 | 0.07 | 0.01 | 0.02 | 0.08 | 0.05 | 0.00 |
| Acq. | 0.05 | 0.05 | -0.02 | -0.01 | -0.03 | 0.08 | -0.01 | -0.02 | 0.00 | 0.00 | 0.01 | 0.03 | 0.02 | 0.08 |

*Notes*. Self indicates continuous self-reported behavior. 'Friend' shows the average of the binned self-reports of friends, 'acq.' that of acquaintances, respectively. 'Perc.' stands for the means of the perceived norm distributions of acquaintances ('acq.') or the population ('pop.').*$p$ < 0.05, **$p$ < 0.01, ***$p$ < 0.001. The Coleman Index is determined according to Signorile & O'Shea (1965) as normalized difference of the fraction of nominations observed to nominations expected by chance for median-split data averaged across both higher and lower half and ranging from full hetrophily at -1 to full homophily at 1.

**S1 Fig .** *Projection effects for selected behaviors at t0.*

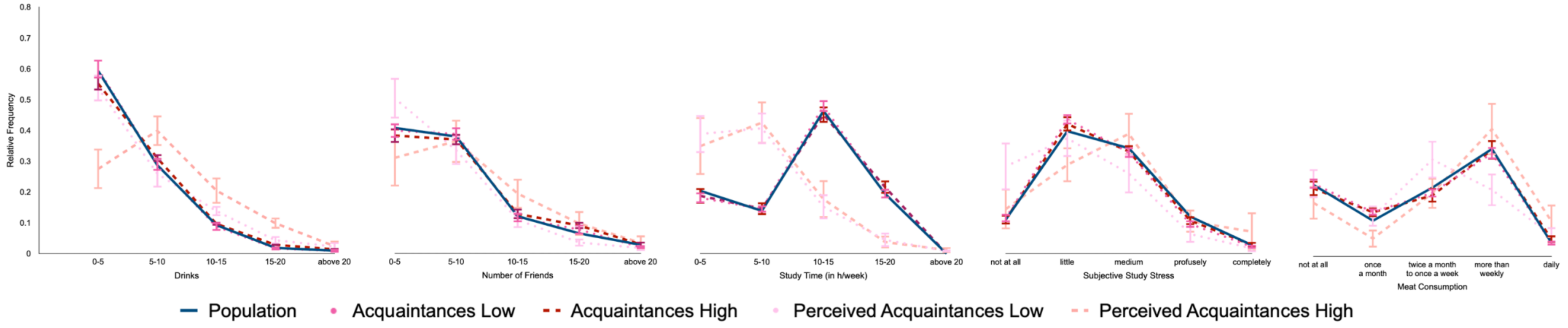


*Notes.* Bars represent 95%CI. Acquaintances of participants with low self-reported behavior (determined by median-splits; Acquaintances Low) do not report lower scores on a behavior than acquaintances of participants with high self-reported behavior (Acquaintances High). However, participants with low and high self-reported behavior perceive the behavior of their acquaintances differently. Participants with low self-reported behavior perceive acquaintances to have a lower distribution (Perceived Acquaintances Low) compared to participants with higher self-reported behavior (Perceived Acquaintances High). These discrepancies cannot be explained by homophilious social circles because the self-reported behavior of acquaintances does not change depending upon the participants' self-reported behavior. If participants selected homophilous acquaintances, one would expect that the acquaintances of the people reporting high values of behavior also report higher values.

## Model estimation and comparison

We compared the unconstrained social sampling model with three parameters (social sampling parameter $\gamma$, behavioral adjustment $\rho$, and memory bias $\alpha$) to nested versions that restricted either one, two, or all three parameters, yielding in total eight different models. Within these nested versions fixed $\alpha$ to 1 (no memory bias), $\rho$ to 0 (no behavioral exclusion) and $\gamma$ to 0 (no social circle). The nested version with all parameters fixed is equivalent to the base rate model that uses the population distribution as a predictor. For all models, the best parameter values were determined by minimizing the deviance, that is $D$ = -2$LL$, $D = -2LL = -2\sum \ln(L)$ twice the negative summed log-likelihood of the model given the data. The likelihood was calculated as the probability of participants' cumulative norm estimates under a normal distribution with the models' predicted cumulative norm estimate as the mean of the normal distribution and a fitted standard deviation $\sigma$. Because we evaluated the likelihood using the cumulative estimates, all models were fitted to $q$-1 response categories for each behavior and participant with one set of parameter values for each participant. Absolute performance of the models was assessed as the percentage of variance explained by the model-predicted norm estimates.

To evaluate the models' relative performance, we employed two model fit indicators that consider different aspects of model complexity and model generalizability: the Bayesian Information Criterion (BIC; Schwarz, 1978) and a cross-validation across time points and across dependent variables, that is a generalization test (Busemeyer & Wang, 2000). These techniques can be used to compare non-nested models, but consider different sources of model flexibility. The BIC penalizes models more harshly that possess a higher number of free parameters $k$, thus accounting for the number of parameters as one source of model flexibility:

$$\mathrm{BIC} = -2LL + k \ln n$$

with $n$ giving the number of data points. BIC weights provide the posterior probability of each model given the data (Wagenmakers & Farrell, 2004).

$$\mathrm{BIC}_{w_M} = \frac{e^{-.5\Delta\mathrm{BIC}_M}}{\sum_i e^{-.5\Delta\mathrm{BIC}_i}}$$

With $\Delta\ \mathrm{BIC}_{\mathrm{M}}$ as the difference between model *M* and the best model in the set and $\Delta\ \mathrm{BIC}i$ as the difference between a specific model *i* and the best model. Differences between two models, $\Delta\mathrm{BIC}_{10} = \mathrm{BIC}_{\mathrm{M1}} - \mathrm{BIC}_{\mathrm{M0}}$, can then be used as well to calculate a Bayes Factor, *BF*, the likelihood of one model M0 compared to an alternative model M1 (Jarosz & Wiley, 2014; Lee & Wagenmakers, 2014):

$$BF_{10} = e^{-\Delta\mathrm{BIC}_{10}/2}$$

**Differentiating the current approach to classic Social Sampling approach**

The classic Social Sampling Model, as defined in Galesic and colleagues (2018), proposes that individuals draw upon the perceived behavior of their acquaintances to infer the population distribution, here the perceived behavior of the entire cohort. In this perceived social circle, individuals similar to oneself may be overrepresented and, therefore, the classic Social Sampling Model then assumes that people exclude individuals who show a similar behavior form their social circle (behavioral adjustment ρ) and may misremember the behavior of their social circle (memory bias α). As such, the classic Social Sampling Model implements the same cognitive adjustment processes as our generalized model, but uses as basis for the population estimation the individual perceptions of the acquaintance distribution, instead of the self-reported behavior of every individual in the population.

We implemented this classic Social Sampling Model (Galesic et al., 2012, 2018) that generalizes the perception of the social circle to the population. Replicating previous work, this classic Social Sampling Model seems to describe population norm perceptions well (Table S2). However, the fit of this Social Sampling Model is only slightly better than the fit of a baseline model that predicts norm perceptions solely from the social circle perception without any corrections. No model is clearly preferred in terms of BICs (Table S2). This baseline model likely fares so well because both pieces of information, the perception of the social circle and the norm perception in the population, are self-reports of the same individual (Table S2). As such, it is unsurprising that predictions based on those self-reports outperform population-based models that are unconflated by similarity in measurement (Table 2). They thereby only illustrate that individual norm perceptions of a subgroup are a good predictor for perceptions of a wider population (and vice versa).

The classic Social Sampling account misses out, however, on explaining how the norm perceptions are established from observations in the social environment. To test the notion that individuals preferably sample information from socially close individuals in their

environment, it is necessary to predict norm perceptions from the social relationships within a social group and independent reports of each person's behavior. In the main study, we relied upon the reported behavior from each individual in the entire cohort, but our generalized model allows to substitute those reports with objective measures of behavior, if available.

**S2 Table.** *Model fits and mean parameter estimates for the classic Social Sampling Model (cSSM)*

| | | | T0 | | | |
|---|---|---|---|---|---|---|
| | $R^2$ | RMSD | BIC | α | ρ | σ |
| Base rate cSSM | 0.81 (0.13) | 14.2 (5.4) | 234 (22) | — | — | 15.9 (6.0) |
| Full cSSM | 0.84 (0.11) | 12.5 (4.3) | 234 (19) | .86 (.14) | .14 (.23) | 14.0 (4.8) |
| | | | T1 | | | |
| | $R^2$ | RMSD | BIC | α | ρ | σ |
| Base rate SSM | .81 (.15) | 13.6 (5.7) | 231 (23) | | | 15.2 (6.3) |
| Full cSSM | .84 (.13) | 11.9 (4.4) | 231 (20) | .88 (.15) | .12 (.21) | 13.3 (4.9) |

*Notes*. Standard deviations in parenthesis. RMSD = Root Mean Square Deviation, BIC =Bayesian Information Criterion, α = memory bias (fixed: α = 1), $\rho$ = behavioral adjustment (fixed: $\rho$ = 0), σ = normally distributed error.

**S3 Table.** *Cross-validation for each model predicting population perception at t1 with model weights of t0.*

| | RMSD | SD | $R^2$ | SD |
|---|---|---|---|---|
| Base Rate | 18.8 | 5.1 | .66 | .15 |
| Memory error (Free α) | 19.6 | 5.3 | .66 | .15 |
| Social Sampling (Free γ) | 18.6 | 5.0 | .66 | .14 |
| Exclude Behavior (Free ρ) | 19.2 | 5.3 | .64 | .15 |
| No memory error (Fixed α) | 19.8 | 5.5 | .64 | .15 |
| No Social Sampling (Fixed γ) | 19.8 | 6.0 | .65 | .15 |
| No Exclude Behavior (Fixed ρ) | 18.6 | 5.2 | .67 | .15 |
| All parameters | 20.5 | 6.5 | .63 | .17 |
